# Neutron Measurements and the Weak Nucleon-Nucleon Interaction

**DOI:** 10.6028/jres.110.023

**Published:** 2005-06-01

**Authors:** W. M. Snow

**Affiliations:** Indiana University/Indiana University Cyclotron Facility Bloomington, IN 47408

**Keywords:** anapole moment, effective field theory, few-body systems, neutral currents, parity violation, QCD, strong interaction, weak interaction

## Abstract

The weak interaction between nucleons remains one of the most poorly-understood sectors of the Standard Model. A quantitative description of this interaction is needed to understand weak interaction phenomena in atomic, nuclear, and hadronic systems. This paper summarizes briefly what is known about the weak nucleon-nucleon interaction, tries to place this phenomenon in the context of other studies of the weak and strong interactions, and outlines a set of measurements involving low energy neutrons which can lead to significant experimental progress.

## 1. Introduction and Discussion

Despite nearly 40 years of study, the details of the weak interaction between nucleons are not understood. This is mainly due to a paucity of experimental results that can be robustly compared with theory. The extreme “weakness” of this interaction implies that it is only experimentally accessible through the study of the measurement of small parity-odd interference effects amid the much larger effects of the strong interaction, described, described by quantum chromodynamics (QCD). Since QCD is a purely vector theory it conserves parity, and so any parity-odd effects must come from the weak interaction. However the natural scale for the size of parity-odd amplitudes, set by the ratio of the amplitudes for W and Z exchange to those for meson exchange between nucleons, is extremely small (≈10^−7^), and therein lies the experimental challenge.

It is important to understand the character of weak interactions between nucleons for a number of reasons. If one assumes the electroweak theory is correct, a study of the weak nucleon-nucleon (NN) interaction has the potential to improve our understanding of the strongly interacting limit of quantum chromodynamics (QCD), which is clearly a problem of fundamental importance. Like the electromagnetic interaction, the weak interaction between quarks and leptons is understood at the fundamental level and is weak enough to probe strongly interacting systems without affecting the strong dynamics. Unlike the electromagnetic interaction, the range of the weak interaction among the quarks, set by the masses of the W and Z bosons, is much smaller than the size of the nucleon as set by the dynamics of the strongly interacting limit of QCD. At the same time the strong repulsion of two nucleons at short distances, understood qualitatively in terms of the Fermi statistics of the quarks in the nucleons and the high energy cost of flipping a quark spin in the nucleon, means that the dynamical mechanism for the weak interaction between nucleons must involve meson exchange and the soft QCD physics that leads to it. We therefore expect that the weak interaction has the potential to provide qualitatively new information on quark-quark correlations in the strongly interacting ground state of QCD and on the underlying physics behind the meson exchange model of the NN interaction.

A physical understanding of the ground state of QCD in the strongly interacting limit does not exist. Such an understanding is one of the goals of the rich field of hadron physics which has opened up between “traditional” nuclear physics and high energy collider physics and which tests the perturbative limit of QCD. As we look to the future beyond the Standard Model (SM), most theorists anticipate that the theory that the SM is embedded in (technicolor, supersymmetry) will possess new strongly interacting sectors. From a theoretical point of view, strong coupling is a phenomenon that is now understood to be the generic norm in quantum field theories rather than the exception. We already know that collective, nonlinear effects are present in the QCD ground state. The approximate chiral symmetry of QCD and the successful interpretation of pions as the low energy excitations of this broken chiral symmetry in the ground state was the first example. The qualitative understanding of many aspects of the QCD vacuum and lattice simulations in terms of helicity-flipping, quark-quark interactions induced by instantons or other nontrivial gluon field configurations is another intriguing possibility. It has also been shown that color superconducting phases caused by BCS-like, quark-quark correlations can exist in the high-density limit of QCD. The ground state of QCD is best thought of as a many-body system for which knowledge of both the elementary excitations of the system and their correlations in the ground state are essential components for a physical understanding. The weak interactions between quarks in nucleons quietly probe the quark-quark correlations and give us a new opportunity to learn about QCD.

The NN weak interaction is also the only practical way to study quark-quark neutral currents at low energy. The neutral weak current conserves quark flavor to high accuracy in the standard electroweak model (due to the GIM mechanism). Therefore it is not seen at all in the well-studied strangeness-changing nonleptonic weak decays. We therefore know nothing experimentally about how QCD modifies weak neutral currents. The effects of quark-quark neutral currents have been seen in collider experiments [1] and the SM predictions have been verified at the high momentum transfers reached in these experiments. Therefore, we have confidence that any deviations from the perturbative QCD predictions for quark-quark neutral currents must be due to strong QCD effects.

With experimental information on the low energy parity-violating (PV) partial waves in the NN system, there is a chance to understand quantitatively for the first time the extensive observations performed in many systems of parity-violating phenomena in nuclei and to use this data to deepen our understanding of nuclear physics. For example, PV can give information on small components of the nuclear wavefunction. In the nuclear shell model, successive levels alternate in parity, and so parity eigenstates are linear combinations of either even (|*nhω* >, *n* even) or odd (|*nhω* >, *n* odd) shell states. Since parity violation directly connects these two subspaces, PV observables are linearly sensitive to small components of the nuclear eigenstates from higher shells [2]. There are a number of observations of PV in parity doublets in nuclei with *A*≈20 that are amenable to a shell model treatment which should be calculable when the PV NN interaction is determined. In addition, ideas from quantum chaos and nuclear statistical spectroscopy have been used to analyze parity violation in neutron reactions in heavy nuclei in terms of the effective isovector and isoscalar weak NN interaction, and knowledge of PV in the NN system would allow a quantitative test of the predictive power of these interesting ideas [3,4]. In both cases, the PV observables open a new window into specific features of the nuclear many-body wave function which can only be exploited if the NN weak interaction amplitudes are known.

NN parity violation also may be relevant to properly interpret certain recent and planned measurements involving PV with electrons. In atoms, the effect of NN parity violation has been seen recently for the first time in ^133^Cs [5] through its contribution to the anapole moment of the nucleus, which is an axial vector coupling of the photon to the nucleus induced mainly by the PV NN interaction [6,7]. Anapole moment measurements in other atoms are possible, and experiments are under way [8]. In the heavy nuclei for which the anapole moment is a well-defined observable, the main contribution to the anapole moment comes from PV admixtures in the nuclear ground state wave function. Although the main contribution to the nuclear anapole comes from the unpaired valence nucleons as in the case of nuclear magnetic moments, one also expects the effects of NN correlations to modify these single-particle estimates [9]. In electron scattering from nucleons, PV effects are sensitive to both Z exchange between the electron and the quarks in the nucleon as well as the coupling of the virtual photon to the axial current from PV interactions among the quarks in the nucleon. A number of experiments to measure PV in electron scattering in different kinematic regions (SAMPLE, HAPPEX, PVA4, G0) will be able to isolate different PV mechanisms and separate out the contribution from q-q weak interactions [10]. Finally, there have been a number of recent calculations of PV effects in Compton scattering [11] and pion photoproduction [12] which are sensitive to q-q weak interactions in the nucleon. As PV effects in electromagnetic processes are increasingly used to learn new information about the nucleon, it will be important to compare the results with the similar processes which contribute to NN parity violation.

Finally there is the question how to describe NN parity violation starting from the Standard Model. The first serious general attempt, and still the most complete theoretical effort in the field, was the work of Desplanques, Donoghue, and Holstein (DDH) [13] and later updates [14]. In this approach, the authors used a valence quark model in combination with SU(6) symmetry relations and data on hyperon decays to produce a range of predictions for effective PV meson-nucleon couplings consistent with the SM. At low energy, the weak interaction between nucleons in this approach is parameterized by the weak pion coupling constant *f*_π_, and six other meson coupling denoted as $h_{\rho }^{0}$, $h_{\rho }^{1}$, $h\prime_{\rho }^{1}$, $h_{\rho }^{2}$, $h_{\omega }^{0}$, and $h_{\omega }^{1}$, where the subscript denotes the exchange meson and the superscript indicates the isospin change. Due to uncertainties in the effects of strong QCD, the range of predictions is rather broad. The types of contributions to the PV NN-meson vertex were identified in this work as (a) the “factorization” process in which the vertex factorizes into a product of a vector (axial vector) N-N current and an axial vector (vector) vacuum-meson current, (b) PV admixtures of the initial and final nucleon states, and (c) contributions from sea quarks. It is interesting that the sign of the p-p and p-^4^He data already implies that contribution (a) is insufficient to describe the PV vector meson couplings. For the weak pion coupling, neutral currents are estimated to play a dominant role. There is no clear subprocess which seems to dominate the total amplitude, but there is speculation that strange quarks [15] and sea quarks [16] play an important role. Another approach is to perform a systematic analysis of the weak NN interaction using an effective field theory approach and chiral perturbation theory to classify the interaction in a manner that is consistent with the symmetries of QCD and does not assume any specific dynamical mechanism. This approach is under construction [17]. However, already an interesting observation has been made with regard to the weak pion coupling: chiral loop effects not included in DDH can have a significant effect on the size of this coupling [18]. In particular, the chiral analysis implies that there are significant contributions associated with disconnected sea quark loops. This is interesting because there are few probes of the u and d sea quark component of the QCD ground state in the non-perturbative regime. Finally, preparations have been made for an eventual calculation of the weak NN interaction vertices using lattice gauge theory in the partially quenched approximation that will undoubtedly be used at first [19]. Although none of these theoretical efforts have yet evolved to produce quantitative predictions for the weak NN interaction directly from the Standard Model, they show that this interaction is indeed sensitive to interesting aspects of strong QCD.

Unfortunately, the rate of progress in our understanding of the NN weak interaction has been slow despite strong experimental activity. Reviews of the subject [9,20,21] reach essentially the same conclusion: the weak NN couplings are unknown. The reasons for the slow advance are both theoretical and experimental. The experimental problems stem from the small size of weak amplitudes relative to strong amplitudes (typically ≈10^−7^ at low energies). The theoretical difficulties are encountered in trying to relate the underlying electroweak currents to low-energy observables in the strongly interacting regime of QCD. One expects the strong repulsion in the NN interaction to keep the nucleons too far apart for a simple direct exchange of W and Z bosons between quarks in different nucleons to represent an accurate dynamical mechanism.

The current approach is to split the problem into two parts. The first step is to map QCD to an effective theory expressed in terms of the important degrees of freedom of low energy QCD, mesons and nucleons. In this process, the effects of quark-quark weak currents appear as parity-violating meson-nucleon couplings [13]. A meson-exchange model is known to work well as a low-energy description of the strong interaction [22]. Even if it is not an honest representation of the NN interaction at the quark level, it at least is a convenient way of encoding the amplitudes. The second step is to use this effective theory to calculate electroweak effects in the NN interaction and to determine the weak couplings from experiment. If the values of the couplings inferred from different experiments are consistent, we can use the results with confidence to improve our understanding of nuclear parity violation. If the meson exchange model fails, then we have learned something interesting about the strongly interacting limit of QCD which would demand explanation.

There are a few general statements that apply to the low energy weak interactions of neutrons with low A nuclei. First of all, there is the question of which observables are nonzero in the limit of zero neutron energy where the experiments can be done. In the absence of resonances it can be shown that the PV helicity dependence of the total cross section vanishes if only elastic scattering is present and that both the PV neutron spin rotation and the PV helicity dependence of the total cross section with inelastic channels are constant in the limit of zero neutron energy [23]. These results depend only on the requirement for parity violation in an S→P transition amplitude involving two-body channels. In the case of PV neutron spin rotation, for example, the PV component of the forward scattering amplitude is proportional to the neutron momentum *k*, and, therefore, the relative phase *ϕ* = (*n* − 1)*kz* between the two neutron helicity states that leads to the rotation after a distance *z*, with *n* = 1 − 2π*f*/*k*^2^ the neutron index of refraction, clearly leads to a nonzero rotation of the plane of polarization per unit length d*ϕ*/d*z*. On the other hand, the PV total cross section difference for elastic scattering is proportional to the imaginary part of the same PV component of the forward-scattering amplitude, and from the optical theorem it follows that *Im*(*f*)/*Re*(*f*) ≈ *kRe*(*f*), which is smaller by an extra factor of *k* and therefore the elastic contribution to the PV cross section difference vanishes. Coupled with the relative ease of polarizing and flipping low-energy neutrons, the inefficiency of MeV gamma polarimeters, and the difficulties of constructing large polarized targets with rapidly reversible polarizations, the two practical classes of neutron PV experiments are (1) PV neutron spin rotation and (2) PV gamma asymmetries.

It is also possible to specify from a purely phenomenological point of view what can be learned about PV NN scattering amplitudes with low-energy neutrons. At the low energies accessible with cold neutrons with *k*_n_*R*_strong_≪1, parity-odd effects in the two-nucleon system can be parameterized by the five independent amplitudes for S-P transitions involving the following nucleons and isospin exchanges: ^1^S_0_ → ^3^P_0_ (pp,pn,nn, Δ*I* = 0,1,2), ^3^S_1_ → ^1^P_1_ (np, Δ*I* = 0), and ^3^S_1_ → ^3^P_1_ (np, Δ*I* = 1). Therefore, from the point of view of a phenomenological description of the weak NN interaction, at least five independent experiments are required. The PV longitudinal analyzing power in pp scattering, which determines a linear combination of the ^1^S_0_ → ^3^P_0_ amplitudes, has been measured at 15 and 45 MeV in several experiments with consistent results [24–27] and remains the only nonzero observation of parity violation in the pure NN system dominated by p-waves. Two of the experiments that are now practical with low-energy neutrons, the PV asymmetry in n-p capture and PV n-p spin rotation, are NN measurements mainly sensitive to the ^3^S_1_ → ^3^P_1_ amplitude. Finally, a search for PV in the helicity dependence of the deuteron photodisintegration cross section, which is becoming practical with the development of the high intensity gamma source (HIGS) laser backscattering gamma facility at the Triangle Universities Nuclear Laboratory (TUNL), would be sensitive to the ^1^S_0_ → ^3^P_0_ and ^3^S_1_ → ^1^P_1_ amplitudes. Therefore, it is possible to foresee important information on all of the PV partial waves coming directly from NN experiments.

In addition to direct measurements of PV in the NN interaction, PV in nuclear few-body systems is also calculable in terms of the PV NN interaction to the required accuracy. In the case of the strong NN interaction, the recent development of Green’s Function Monte Carlo (GFMC) techniques, coupled with the extensive strong NN interaction database, has allowed the energy levels of light (*A*<6) nuclei to be calculated to ≈1 % uncertainty. With the strong interaction contribution to nuclear wave functions known, the effects of the weak NN interaction can be added in as perturbations to see how they contribute to PV observables in finite nuclei. These calculations have recently been done for np and pp parity violation [28,29,30] and can be done in principle for all light nuclei. Therefore, the nonzero PV effect observed already in the p-^4^He analyzing power at 46 MeV [31] can be used with confidence to determine the PV NN interaction. Two more experiments, now practical with low-energy neutrons, the PV asymmetry in n-D capture and PV n-^4^He spin rotation, can also be cleanly interpreted. Therefore, the additional four PV NN experiments that can be performed with low-energy neutrons have the potential to qualitatively transform our knowledge in this area. It is also possible that these microscopic calculations can also be applied in the near future to systems with somewhat larger A, such as ^10^B and ^6^Li, where measurements of P-odd observables with low energy neutrons is also possible [32].

**Fig. 1:**
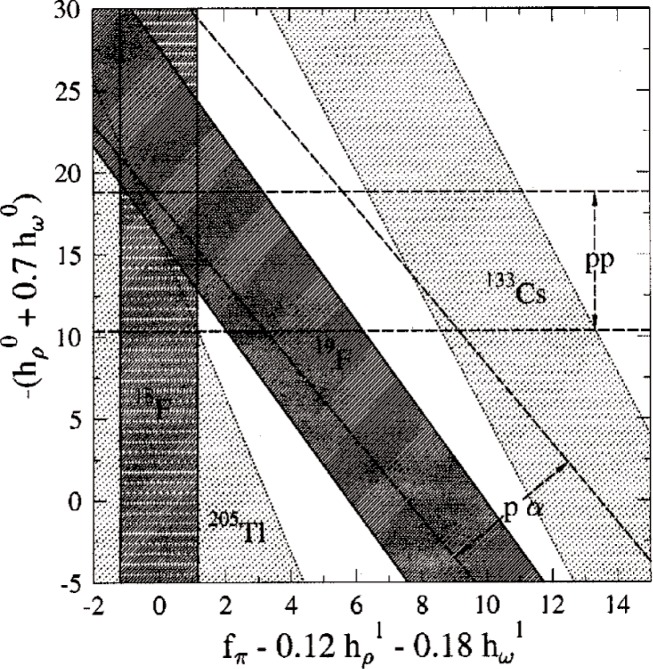
Constraints on linear combinations of isoscalar and isovector nucleon-nucleon weak meson couplings [from W. C. Haxton, C.-P. Liu, and M. J. Ramsey-Musolf, Phys. Rev. Lett. 5247 (2001)].

The longest-range part of the interaction is dominated by pion-nucleon coupling constant *f*_π_. Measurements of the circular polarization of photons in the decay of ^18^F provide a value for *f*_π_ that is considerablye smaller than the DDH “best value” though still within the reasonable range. A precision atomic physics measurement of the ^133^Cs hyperfine structure (anapole moment) has been analyzed to give a collective constraint on *f*_π_ and the combination $\left(h_{\rho }^{0}+0.6\;h_{\omega }^{0}\right)$. This result would seem to favor a value for *f*_π_ that is inconsistently larger than the ^18^F result (though still just within the DDH reasonable range). Figure 1 presents an exclusion plot that summarizes the current situation.

There are four plausible experiments which employ beams of cold neutrons and involve targets with *A*<5: measurement of the PNC gamma asymmetries in n + p → d + γ and in n + D → t + γ and of the PNC neutron spin rotations in ^4^He and H_2_. An attractive feature of the neutron experiments is their differing sensitivity to the different meson couplings. Table 1 [20] gives an estimate of the contribution to the PNC observable from each meson coupling.

It is clear from this table that a measurement of the weak pion coupling is required to extract information on the other weak couplings from neutron measurements. Because of their reduced sensitivity to the ρ and ω couplings, the gamma asymmetry in n + p → d + γ and the n-p spin rotation experiment provide an excellent laboratory for the determination of *f*π. In the n-^4^ He spin rotation and n + p → t + γ systems, the isoscalar ρ coupling has about the same size relative to the isovector π coupling but has opposite signs, so a comparison of these two results with each other and with the p-^4^He result would be a good way to isolate the isoscalar ρ coupling. With p-p scattering there is accurate information on a linear combination of ρ and ω couplings, and both the n-^4^He and n + p → t + γ measurements are also sensitive to ω couplings.

From these comparisons, it should be clear that neutron measurements can have a strong impact on the NN weak interaction field. If the precision of these measurements is set only by the statistical sensitivity achievable at the Spallation Neutron Source under construction at Oak Ridge National Lab, for example, then 4 of these couplings can be determined to better than 30 % accuracy [39]. Experiments in progress to search for the gamma asymmetry in n + p → d + γ at LANSCE and parity-odd spin rotation in n-^4^He at NIST and plans for the measurement of PV spin rotation in n-p are described in other contributions in this issue.

## Figures and Tables

**Table 1:** Expansion coefficients for the contributions to the PNC observables from the individual meson-exchanges and calculated observables for PV NN observables that are either already measured (p-p and p-^4^He) or proposed (neutron measurements). Calculations in Table 1 for the neutron observables are from [30] for n-p spin rotation, [34,35] for n-^4^He spin rotation, [29,33,36–38] for n-p capture, and [40,41] for n-D capture

	n+p→d+γ	np spin rotation	n+d→t+γ	nα^4^He spin rotation	p-p scattering	p-^4^He scattering
*f*_π_	−0.107	−3.12	0.92	−0.97		−0.340
$h_{\rho }^{0}$		−0.23	−0.50	−0.32	0.079	0.140
$h_{\rho }^{1}$	−0.001		0.103	0.11	0.079	0.047
$h_{\rho }^{2}$		−0.25	0.053		0.032	
$h_{\omega }^{0}$		−0.23	−0.160	−0.22	0.073	0.059
$h_{\omega }^{1}$	0.003		0.002	0.22	0.073	0.059
